# Quasi-Experimental Study Assessing the Effectiveness of an Educational Intervention for Fall Prevention Among Older Adults in Saudi Arabia

**DOI:** 10.3390/healthcare14121771

**Published:** 2026-06-19

**Authors:** Anwar Alhashem, Reham Alharbi, Rayouf Al-Otaibi, Nora Alsakran, Aryam Alharbi, Ghaida Hakami

**Affiliations:** Department of Health Sciences, College of Health and Rehabilitation Sciences, Princess Nourah bint Abdulrahman University, P.O. Box 84428, Riyadh 11671, Saudi Arabia; amalhashem@pnu.edu.sa (A.A.); reoufx1166@gmail.com (R.A.-O.); norah.alsakran2003@gmail.com (N.A.); 197aryam@gmail.com (A.A.); ghaida3299@gmail.com (G.H.)

**Keywords:** Stopping Elderly Accidents, Deaths, & Injuries, fall prevention, knowledge, skill

## Abstract

**Highlights:**

**What are the main findings?**

- The Arabic adaptation of the STEADI-based educational program was associated with short-term improvements in fall prevention knowledge, practical skills, and behavioral intentions among older adults.

**What are the implications of the main findings?**

- The results support the integration of fall prevention programs in community clubs and social services.
- Interventions should incorporate both cognitive and skill components, which may support short-term improvements in fall prevention among older adults.

**Abstract:**

**Background:** With increasing life expectancy, older adult populations worldwide are growing rapidly. Falls are among the most prominent problems that older adults face. This study aimed to assess the educational components of the Stopping Elderly Accidents, Deaths, & Injuries (STEADI) program for improving knowledge, skills, and behavioral intentions for fall prevention among older adults. **Methods:** A quasi-experimental study was conducted with a non-equivalent control group pretest–posttest design, involving 128 older women (≥60 years) in a community center in Riyadh. Data were collected using a structured questionnaire. Descriptive statistics were used to summarize the data. Pearson’s chi-square test was performed to compare demographic and physical characteristics between the groups. Independent-sample *t*-tests, effect size calculation (Cohen’s d), and ANCOVA-adjusted analyses were used to compare post-intervention outcomes between groups. Within-group changes were compared using a paired *t*-test. Additionally, one-way analysis of variance (ANOVA) was performed to compare the demographic, health, and physical characteristics of the participants. Statistical significance was set at *p* ≤ 0.05. **Results:** The intervention group showed improved knowledge (t = 11.654), skills (t = 7.961), and intention to perform preventive behaviors (t = 3.785), with a significant *p*-value of <0.0001. Large intervention effects were observed for knowledge (Cohen’s d = 2.30) and skills (Cohen’s d = 1.57). ANCOVA-adjusted analyses confirmed significant intervention effects for knowledge (adjusted mean difference = 5.06, 95% CI 4.46–5.66, *p* < 0.001) and skills (adjusted mean difference = 1.87, 95% CI 1.56–2.18, *p* < 0.001). **Conclusions:** The results indicate that the STEADI program produces significant short-term improvements in knowledge, skills, and behavioral intentions related to fall prevention. The findings emphasize the importance of integrating prevention programs into community settings and activating the role of families in supporting preventive practices.

## 1. Introduction

Falls are defined as unexpected events that result in a person coming to rest accidentally on the floor or at a lower level [1]. Globally, falls are a major public health issue owing to their association with injuries, disabilities, and increased dependency among older adults. Worldwide, annually, approximately 37.3 million falls result in serious injuries or disabilities that require medical attention, causing a loss of more than 38 million disability-adjusted life years (DALYs) [2]. In a systematic review and meta-analysis of 104 studies, the global prevalence of falls was found to be 26.5%. Specifically focusing on the older population in Asia, data from 48 of these studies revealed a fall prevalence of 25.8% [3]. Older adults aged 65 years and above are the most vulnerable to falls [4]. Hospitalization due to falls often results in significant healthcare costs and increased morbidity among affected individuals. In the Netherlands, fall-related injuries in older adults have led to an estimated annual healthcare cost of approximately $512.6 million, accounting for 20.8% of the national healthcare budget for injuries. Hip fractures are the most significant contributor, accounting for 42.3% ($220.50 million USD) [5].

Falls in Saudi Arabia represent a growing public health challenge as the Kingdom experiences a significant increase in its aging population [6]. According to reports published by the General Authority for Statistics, older adults represented approximately 2.7% of the Saudi Arabian population in 2022, increasing to 4.8% in 2025 [7,8]. These demographic changes highlight the growing aging population.

Studies in Saudi Arabia report that 44.2–57.7% of older adults have experienced at least one fall. In Riyadh, Saudi Arabia, fall prevalence was 57.7% among older adults aged 80–89 years; 81.7% of them had a fall due to environmental factors, including cluttered pathways, poor lighting, uneven floor surfaces, unsecured carpets, and elevated door sills [9]. In Unaizah, a central-northern city in Saudi Arabia, a study found that fall prevalence in 2018 was 31.6%, with women experiencing more falls than men (34.5% vs. 28.5%) [10]. Therefore, meeting the health needs of aging populations is becoming an increasingly urgent public health priority [3].

Balance impairment is the primary factor contributing to falls among older adults. Older adults with impaired gait show higher fall risk rates (83.3%) [9], attributed to the decline in efficiency of the musculoskeletal, central nervous, and sensory systems [11]. Medication use is another critical factor associated with fall risk in older adults. There is a strong correlation between taking more than four medications and an increased risk of falls, particularly with psychotropic drugs such as antidepressants, sedatives, hypnotics, and benzodiazepines. These medications can cause side effects or interactions that contribute to instability and falls [12].

Educational interventions aimed at fall prevention have increasingly been acknowledged as cost-effective strategies for mitigating fall risk and promoting healthy aging among older adults residing in community settings. Numerous studies have demonstrated that such educational interventions can enhance knowledge, awareness, self-efficacy, and engagement in preventive behaviors related to home safety, physical activity, and environmental modification [12,13,14,15,16]. One pilot study evaluating a health belief model—based on educational intervention among older adults receiving physical therapy—reported significant improvements in fall risk knowledge and awareness of medication-related fall risks, with awareness improving by 50% following the intervention. In addition, 75% of participants identified feasible home modifications, 62% reported motivation to implement prevention strategies, and 50% adopted lifestyle changes following the educational session. Notably, only one fall was reported during the 60-day follow-up period compared with four falls reported during the previous two years, suggesting that structured educational interventions may contribute to lowering the risk of falls among older adults [17]. Similarly, a randomized controlled trial evaluating a home hazard modification program demonstrated reductions in falls, including an 18.5% reduction in overall falls and a 13.2% reduction in indoor falls within 12 weeks following the intervention, particularly among individuals aged ≥75 years [12]. Furthermore, tailored multimedia-based educational interventions have demonstrated improvements in fall prevention knowledge and behavioral engagement among older adults. One randomized trial reported significantly greater knowledge gains among intervention participants compared with controls (*p* = 0.004 and *p* = 0.002), while 94.3% of participants adopted at least one new fall prevention strategy following the intervention [14]. Collectively, these findings support the integration of educational and community-based fall prevention interventions as evidence-based strategies for improving safety awareness and reducing fall-related morbidity among older adults. In Saudi Arabia, fall prevention education has increasingly emerged as a public health priority. The Ministry of Health has introduced national guidelines for fall prevention and management, alongside educational initiatives aimed at healthcare providers and older adults [18]. Concurrently, the Saudi Patient Safety Center has developed educational resources and practical guidance concerning falls and fall prevention in adults. These initiatives aim to enhance awareness of fall risks and promote evidence-based prevention strategies within healthcare and community settings. The resources are designed to support older adults, caregivers, and healthcare providers by enhancing their understanding of environmental safety, mobility support, the role of medications in increasing fall risk, and other preventive measures [19].

The Stopping Elderly Accidents, Deaths, & Injuries (STEADI) initiative, developed by the Centers for Disease Control and Prevention (CDC), serves as a comprehensive framework for healthcare providers to identify, assess, and intervene in evidence-based practice to manage fall risk among older adults [13]. Several studies have documented the successful implementation of the STEADI initiative within clinical settings. In Portland, Oregon, a study conducted over six months involved 18 healthcare providers. During this period, 773 screenings of older adults were conducted, and 22% were identified as being at high risk of falls. The majority of them received interventions, including gait assessments, vision evaluations, vitamin D reviews, and foot assessments [14]. Another study conducted between 2017 and 2021 found that STEADI is an effective initiative for reaching older adults at risk of falls in outpatient rehabilitation settings. In a screening of over 50,000 older adults across 34 outpatient rehabilitation clinics in the USA, 76.4% were assessed for fall risk, with 44.1% identified as being at risk for falls [15].

Numerous studies have used the STEADI initiative to screen and identify individuals at risk of falls. To the best of our knowledge, there is a paucity of research examining the educational components of the STEADI initiative in Arab community-based contexts. Consequently, this study employed STEADI strategies for fall prevention, including postural hypotension, chair exercises, safe shoes, and the My Medication List, which were available on the STEADI educational website, brochures, booklets, videos, and factsheets. While the STEADI initiative was initially developed in the United States, it is crucial to adapt fall prevention educational materials to the Saudi Arabian and broader Arab contexts because of differences in language and culture. A significant number of older adults in Saudi Arabia primarily communicate in Arabic and may possess varying levels of health literacy, necessitating culturally appropriate Arabic educational materials for effective communication and engagement. This study involved a fall prevention educational program delivered in Arabic targeting older adults in a community center setting. It aimed to assess short-term improvements in fall prevention knowledge, skills, and behaviors among older adults in Riyadh.

## 2. Materials and Methods

### 2.1. Educational Framework

In this study, the researchers developed a fall prevention education program based only on resources from the STEADI website, brochures, booklets, videos, and fact sheets. The program emphasizes the following key topics: fall prevention, postural hypotension, foot care and footwear for older adults, chair-rise exercises, and medication education. The translated materials were organized into a structured educational session. The program comprised ten sequential segments delivered over approximately 2 h. It began with an introductory overview of the session, followed by general information on falls. Subsequent segments addressed the risk factors and consequences of falls, as well as essential fall prevention strategies at home. A short break was provided midway through the session. It concluded with a summary and key take-home message.

Since the original STEADI educational materials were developed in English, the research team translated the materials into Arabic. The translated version was subsequently reviewed, corrected, and approved by a certified English–Arabic language specialist. The final Arabic version was further reviewed by an Arabic language expert to ensure linguistic accuracy, clarity, and cultural appropriateness for the target population.

### 2.2. Study Design and Setting

This study utilized a quasi-experimental design with a non-equivalent control group pretest–posttest method. Ethical approval was obtained in January 2025. The educational program and data collection were conducted over two months, from February to March 2025, at a community center in Riyadh.

### 2.3. Participants and Sampling

The sample size was calculated using G*Power software v3.1.9.7 (Düsseldorf, North Rhine-Westphalia, Germany) for a two-tailed test comparing two independent means (two groups). The parameters included an effect size (d) of 0.5, a significance level (α) of 0.05, statistical power (1 − β) of 0.80, and an allocation ratio (N2/N1) of 1.

This resulted in the required sample size of 128 participants, with 64 participants in each of the intervention and control groups. Participants were recruited through convenience sampling and assigned to intervention and control groups based on their willingness to participate. Randomization was not feasible in the study setting because of practical and logistical constraints inherent in the community center environment. Many older adult participants attended the center accompanied by friends or family members, complicating group separation during recruitment and intervention delivery.

Given the non-random group allocation, this study was subject to potential self-selection bias, as participants who agreed to participate in the intervention may have been more motivated or receptive to educational activities than those in the control group.

The study included participants aged 60 years and above with intact cognitive abilities. Individuals with cognitive impairment were excluded, as this study aimed to assess changes in knowledge following the educational program. Cognitive impairment was evaluated through the researchers’ observations and brief interactions with the participants during the recruitment process. No participants exhibited observable indicators of significant cognitive impairment during recruitment, data collection, or the educational intervention.

In addition, participants with mobility limitations (motor impairments) were excluded to ensure they could safely perform the chair-rise exercise component of the intervention. Participants who did not speak Arabic were also excluded.

### 2.4. Data Collection

The research team collected data from both the control and intervention groups using an interviewer-administered questionnaire, with responses recorded directly in a Google Forms survey. Data collection and intervention delivery were executed by a team of five trained researchers, with responsibilities allocated across various study procedures. This distribution likely contributed to minimizing potential assessment bias. Formal interrater reliability was not performed. The researchers responsible for delivering the intervention in a particular session did not conduct outcome assessments during the same session; instead, assessment duties were rotated among team members in subsequent sessions. The researcher read each question to the participants and recorded their responses accordingly. For the skill domain, the participants were asked to identify safe and unsafe footwear from a predefined set of images derived from STEADI materials. In addition, chair-rise exercise skills were assessed through direct observation following the educational session using the STEADI chair-rise exercise instructions. Participants were asked to perform the chair-rise exercise once under the supervision of a researcher using a stable chair. Performance was evaluated using an observational rating scale categorized as excellent, acceptable, or poor based on the participants’ ability to correctly perform the demonstrated movements and follow the exercise instructions. Safety precautions included researcher supervision throughout the exercise and immediate discontinuation if participants experienced discomfort or instability.

### 2.5. Study Instrument

We used a structured questionnaire adapted from the CDC’s STEADI materials. The pre-intervention questionnaire consisted of four parts:

The following socio-demographic and health-related data were collected: age, educational level, self-reported health issues, need for assistance from family or domestic workers/nurses, level of independence in daily tasks, use of walking aids, use of medications, fall history, frequency of falls in the past year, and causes of falls.

The fall prevention knowledge section assessed participants’ comprehension of fall risk factors and prevention strategies. The scale consisted of 15 items with a possible score range of 0–15, where each item was scored as 1 for a correct response and 0 for an incorrect or “I do not know” response. It included items to evaluate awareness of medical-related causes of falls, such as postural hypotension, dehydration, and foot edema. Additional items addressed the impact of foot pain, the importance of regular foot examinations, the characteristics of safe footwear, including proper fit and appropriate heel height, and the timing of shoe purchases. The section also assessed knowledge related to exercise, particularly the benefits of chair exercises for muscle strengthening and how to perform them correctly. Furthermore, the participants were asked about the benefits of medication management and environmental risk factors, including flooring conditions and poor lighting, as potential causes of falls.

The skills section evaluated participants’ proficiency in executing tasks related to fall prevention. The scale consisted of four items with a possible score range of 0–8, where each item was scored as 0 for poor, 1 for acceptable, and 2 for excellent performance. The items assessed included selecting appropriate and safe footwear, identifying unsafe footwear, safely transitioning from a sitting to a standing position, and performing chair-based exercises.

The behavior section encompassed items assessing the extent to which participants engaged in protective versus risky behaviors that may influence their likelihood of falls. The scale consisted of 7 items with a possible score range of 0–14, with response options scored as 0 for “Never,” 1 for “Sometimes,” and 2 for “Always.” Questions included rushing while performing tasks; neglecting to pay attention to environmental hazards, such as failing to notice floor spills; and insufficient caution when walking outdoors. Additional items investigated whether individuals adopted safe practices during routine activities, including being mindful while moving, avoiding risky actions, and maintaining awareness of their surroundings.

The post-intervention version included the exact items on fall prevention knowledge and skills, and only behavioral questions were replaced with behavioral intention items. This approach is consistent with educational intervention studies, where behavioral intention is considered an appropriate proximal outcome reflecting readiness to change and the potential for future behavior adoption. This approach is conceptually supported by the theory of planned behavior, which considers behavioral intention as an important predictor of future health behaviors and readiness to adopt preventive practices. Therefore, the behavioral intention section assessed participants’ intentions to adopt fall prevention behaviors within the next three months. The behavioral intention scale consisted of 7 items with a possible score range of 0–14, with response options scored as 0 for “I do not plan to do that,” 1 for “I am thinking about it,” and 2 for “I plan to do that.” The items examined planned actions such as informing a doctor if they experienced imbalance, bringing and reviewing medications during medical visits, and monitoring the effects of new medication on balance. Additional items assessed aspects such as rearranging home furniture to facilitate safe movement, taking steps to improve home safety, intention to engage in muscle-strengthening exercises, and getting out of bed slowly upon waking. Higher scores across all scales represented more favorable fall prevention outcomes.

The pre–post intervention questions used in this study were constructed based on educational materials provided by the STEADI initiative. The original content was carefully reviewed and used to create Arabic questions that aligned with the study objectives. Three experts evaluated the face validity of the questionnaire, and minor modifications were made to ensure clarity and cultural appropriateness. The panel included one community medicine consultant and two health education consultants.

Internal consistency of the questionnaire was assessed using Cronbach’s alpha. The questionnaire included items measuring knowledge (15 items), skills (four items), behavior (seven items), and behavioral intention (seven items).

The knowledge scale demonstrated acceptable reliability (α = 0.71), and the behavioral intention scale demonstrated good reliability (α = 0.81). Lower reliability coefficients were observed for the skills scale (α = 0.32). Therefore, findings related to this domain should be interpreted cautiously. Although the limited number of items may have contributed to the lower alpha coefficient, future studies should further validate and refine this subscale. Similarly, the behavior subscale demonstrated modest internal consistency (α = 0.50), and findings based on this scale should be interpreted with caution.

### 2.6. Intervention Description

The intervention consisted of a structured educational session delivered in a group setting in a meeting room and a social lounge at the center. The sessions were led by trained researchers who had completed STEADI training and obtained certification from the Public Health Authority. Each older adult participant attended one standardized 2 h session, with approximately 6–8 participants per session. A total of three to five researchers were involved in each session, including researchers responsible for delivering the educational intervention and others assigned to outcome assessment. A STEADI-aligned PowerPoint presentation was used to ensure consistency across sessions during data collection. The same educational materials, presentation structure, and practical demonstrations were used across all sessions to support standardized intervention delivery. The content covered includes postural hypotension, fall prevention strategies, safe footwear selection with visual examples, chair-rise exercises with live demonstrations, and the My Medication List. The session incorporated interactive discussions and practical application of exercises. Participants received printed educational materials in Arabic, including fall prevention tips and the My Medication List card. The participants completed pre- and post-intervention questionnaires during the same session.

### 2.7. Data Analysis

Data were analyzed using SPSS Pc+ 26.0 version (IBM Inc., Chicago, IL, USA). Descriptive statistics (mean, standard deviation, frequencies, and percentages) were used to summarize quantitative and categorical variables. Pearson’s chi-squared test was used to compare the distributions of demographic and physical characteristics between the intervention and control groups. Student’s *t*-tests for independent samples (intervention and control groups) were used to compare the mean values of the quantitative outcome variables (knowledge scores, fall prevention skills, and behavioral intentions). The comparison of mean differences in pre- and post-intervention scores for knowledge and skills, in relation to the demographic, health, and physical characteristics of the intervention group, was carried out using Student’s *t*-test for independent samples and one-way analysis of variance. The mean values of behavioral intention scores after the intervention were compared based on the demographic, health, and physical characteristics of the intervention group using Student’s *t*-test for independent samples and one-way analysis of variance. Effect sizes were calculated using Cohen’s d. ANCOVA models controlling for baseline scores were used to estimate adjusted intervention effects. Within-group pre–post changes in knowledge and skills were examined using paired *t*-tests. Partial eta squared (η^2^) values were reported as measures of effect magnitude. A *p*-value of ≤0.05 was used to report statistical significance.

### 2.8. Ethical Considerations

Ethical approval (IRB 25-0011) was obtained from Princess Nourah bint Abdulrahman University. All the participants provided written informed consent. Participation was voluntary, and no monetary incentives were provided. Participants were informed that their decision to participate or decline participation would not affect their membership status or access to services at the center. Confidentiality was strictly maintained and participants were free to withdraw from the study at any time without any consequences.

## 3. Results

### 3.1. Comparison of Demographic and Health Characteristics Between Intervention and Control Groups

Table 1 presents information on the study sample, which primarily consisted of older adults in the younger age range, with the majority of participants in both the intervention and control groups aged 60 to 69 years (65.6%), while 34.4% were aged 70 to 79 years. Regarding their educational background, a significant proportion of the study population had limited formal education. In the intervention group, most participants either were illiterate (35.9%) or had completed only primary education (20.3%), whereas in the control group, the largest proportion completed primary education (32.8%) or were illiterate (18.8%). Only a small percentage achieved university education or higher, at 7.8% in the intervention group and 17.2% in the control group. Regarding health status, the study population had a high prevalence of chronic conditions, particularly hypertension (55.2% and 41.8% in the intervention and control groups, respectively), diabetes (51.7% and 40.0%, respectively), and musculoskeletal issues (25.9% and 40.0%, respectively). Medication use was also prevalent, with most participants reporting the use of 1–3 medications (60.9% in the intervention group and 48.4% in the control group), whereas 26.6% and 34.4%, respectively, were taking four or more medications. The comparison of all the characteristics between intervention and control groups shows no statistically significant difference. Overall, the findings indicate that the intervention and control groups were comparable at the baseline in terms of demographic and health characteristics.

### 3.2. Comparison of Physical Condition Characteristics Between Intervention and Control Groups

Most participants in both groups did not use walking aids, although a slightly higher proportion was observed in the intervention group (93.8%) than in the control group (82.8%); however, this difference was not statistically significant (*p* = 0.054). Daily support for routine tasks, level of independence, and history of falls were comparable between the two groups, with no statistically significant differences (*p* > 0.05). Among the participants who reported a history of falls, the number of falls in the past year and the cause of the last fall were similar in both groups. Slipping on wet surfaces and tripping over objects were the most frequently reported causes. These findings suggest baseline similarity between the groups (see Table 2).

### 3.3. Comparison of Pre-Intervention Mean Values of Knowledge, Skills, and Behavior Scores

As shown in Table 3, the mean pre-intervention scores for knowledge, skills, and behaviors related to fall prevention were similar between the intervention and control groups. The mean knowledge score was 8.03 ± 3.3 in the intervention group and 8.53 ± 2.7 in the control group, with no statistically significant difference (*p* = 0.346).

Similarly, the mean skills scores (5.44 ± 1.4 vs. 5.28 ± 1.7; *p* = 0.577) and behavior scores (8.53 ± 2.5 vs. 8.25 ± 2.3; *p* = 0.503) did not differ significantly between groups. The 95% confidence intervals for the differences in mean scores included zero for all outcomes, indicating baseline similarity between the two groups before the intervention.

**Figure 1:**
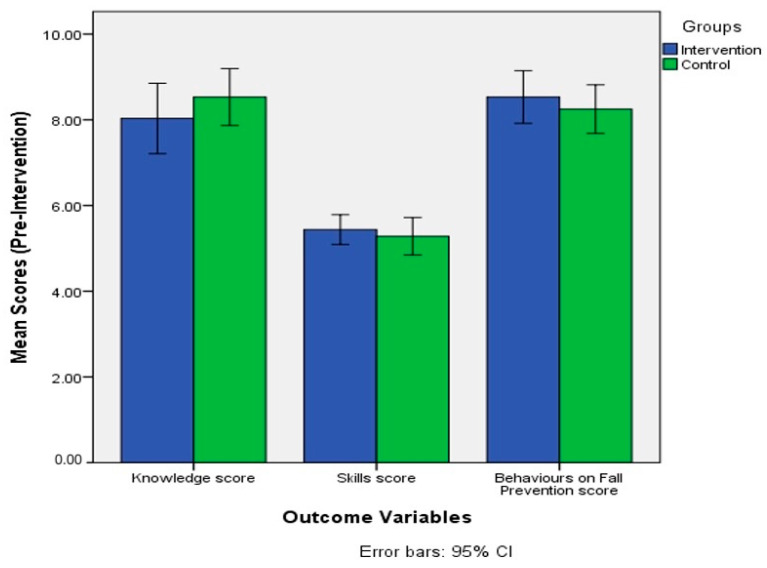
Baseline comparison of outcome scores in the intervention and control groups.

Figure 1 illustrates the comparison of pre-intervention mean scores for knowledge, skills, and behaviors related to fall prevention between the intervention and control groups. The figure shows similar baseline scores across both groups for all three domains, supporting the absence of significant differences prior to the intervention.

### 3.4. Adjusted Post-Intervention Comparison of Outcome Variables Between the Intervention and Control Groups

Table 4 presents ANCOVA-adjusted post-intervention comparisons of outcome variables between the intervention and control groups, together with effect size estimates. Knowledge scores were significantly higher in the intervention group than in the control group (Cohen’s d = 2.30). Skills scores also demonstrated a large effect size (Cohen’s d = 1.57). ANCOVA controlling for baseline values confirmed significant intervention effects for knowledge (adjusted mean difference = 5.06, 95% CI 4.46–5.66, *p* < 0.001, partial η^2^ = 0.73) and skills (adjusted mean difference = 1.87, 95% CI 1.56–2.18, *p* < 0.001, partial η^2^ = 0.58). After adjustment for baseline scores using ANCOVA, the intervention remained significantly associated with higher post-intervention knowledge and skills scores. The intervention effect for knowledge was large, and the effect for skills was also large; however, skills findings should be interpreted cautiously because of the low internal consistency of the skills scale. Behavior intention scores also showed a statistically significant difference, with higher scores observed in the intervention group (13.38 ± 1.6) compared with the control group (11.34 ± 3.3) (*p* < 0.0001).

**Figure 2:**
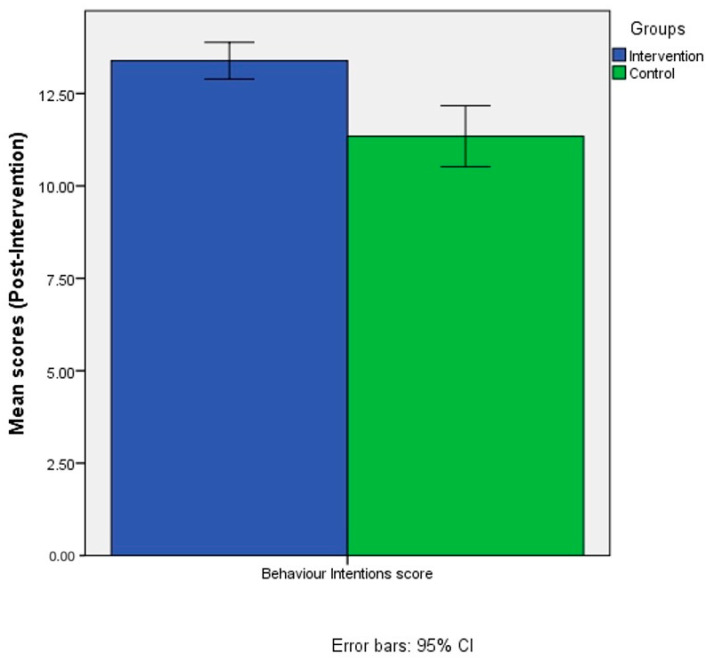
Post-intervention comparison of behavioral intention scores in the intervention and control groups.

Figure 2 presents a comparison of post-intervention mean scores of behavioral intentions between intervention and control groups. The figure shows higher mean scores of behavioral intentions in the intervention group compared with the control group.

### 3.5. Comparison of Mean Differences in Knowledge and Skills Scores Across Characteristics in the Intervention Group

Table 5 summarizes the mean differences between the pre- and post-intervention knowledge and skills scores across selected demographic, health, and physical characteristics within the intervention group. Improvements in knowledge scores were observed across all subgroups. A statistically significant difference in knowledge score improvement was observed with daily support for routine tasks (*p* < 0.0001) and level of independence (*p* = 0.034), with higher knowledge scores observed among participants who relied on family members for support and those who were not independent or partially dependent on themselves for daily tasks. For the other variables (age group, educational level, use of medication, history of falls, and number of falls in the past year), there were no statistically significant differences in mean knowledge scores between pre- and post-intervention.

For skills scores, statistically significant differences were observed for medication use (*p* = 0.028), indicating higher skills scores among participants who had used four or more medications. No significant differences were found for age group, education level, daily support for routine tasks, independence level, history of falls, or number of falls in the past year (*p* > 0.05). Overall, the intervention was associated with short-term improvements in knowledge and skills across some of the participant characteristics in the intervention group.

### 3.6. Comparison of Post-Intervention Behavior Intention Scores Across Characteristics in the Intervention Group

Table 6 presents post-intervention behavior intention scores across demographic, health, and physical characteristics in the intervention group. Mean behavior intention scores were high across all subgroups, indicating a strong intention to adopt fall prevention behaviors following the intervention.

No statistically significant differences were observed based on age, education level, medication use, daily support, independence level, history of falls, or number of falls in the past year (*p* > 0.05 for all comparisons). These findings suggest that the intervention was equally effective in improving behavioral intentions across diverse participant characteristics.

### 3.7. Comparison of Pre- and Post-Intervention Mean Knowledge and Skills Scores in the Intervention Group

Within-group analyses among intervention participants demonstrated significant improvement in knowledge (mean change = 5.21, 95% CI 4.22–6.20, t = 10.59, *p* < 0.001, Cohen’s d = 1.61) and skills (mean change = 1.79, 95% CI 1.38–2.20, t = 8.78, *p* < 0.001, Cohen’s d = 1.34), indicating large educational effects (see Table 7).

## 4. Discussion

This study aimed to implement the STEADI program in Arabic for older adults to enhance their knowledge, skills, and behavioral intentions regarding fall prevention. The results indicated short-term improvements in the intervention group. These findings suggest potential benefits of structured educational interventions in supporting fall prevention awareness and practical safety skills among older adults.

### 4.1. Baseline Characteristics and Fall Risk Profile

The results show no statistically significant differences between the intervention and control groups regarding demographic characteristics, including age and educational level, or in health variables, such as medication use, fall history, causes of falls, and chronic health conditions such as diabetes, hypercholesterolemia, and hypertension. These findings suggest that the two groups were generally comparable at baseline. However, internal validity remains limited due to the non-randomized study design and the potential for self-selection bias. Therefore, the observed post-intervention improvements should be interpreted cautiously.

The study sample primarily consisted of younger older adults, with most participants aged between 60 and 69 years. The educational profile of the participants reflected generally low levels of formal education, with many being illiterate or having completed only primary education. This distribution may indicate potential health literacy challenges and highlights the importance of providing simplified and accessible educational interventions tailored to older adults.

Additionally, the study population had a high prevalence of chronic conditions, particularly hypertension, diabetes, and musculoskeletal disorders. This finding is consistent with previous research indicating that non-communicable diseases increase significantly with age and represent a major health burden among older adults [16]. The presence of multiple chronic conditions may further contribute to an increased risk of falls because comorbidities can negatively affect balance, neuromuscular control, and overall functional ability [17].

Furthermore, medication use was highly prevalent among participants, with many reporting the use of multiple medications. These findings align with existing evidence suggesting a strong association between polypharmacy and increased risk of falls in older adults [18]. Certain categories of medications, particularly antihypertensive and psychotropic medications, and those that affect balance, have been shown to significantly increase fall risk [19]. Collectively, these findings underscore the multifactorial nature of fall risk in this population and highlight the importance of incorporating comprehensive risk education in fall prevention programs.

Functional characteristics of the participants indicated that most older adults did not rely on walking aids, suggesting a relatively preserved level of mobility in the study population. Additionally, varying levels of independence were observed, with some participants requiring assistance in performing daily tasks. These findings reflect a largely mobile population that may still experience functional limitations. Functional indicators, such as the use of walking aids, level of autonomy, and need for daily support, are important determinants of fall risk. Previous studies have shown that functional impairment is one of the strongest predictors of falls among older adults, as it directly affects balance, coordination, and physical stability [17,20].

### 4.2. Effect of the Intervention on Fall Prevention Knowledge

The intervention was associated with a marked short-term improvement in fall prevention knowledge, with scores approximately 5 points higher in the intervention group. The improvement in knowledge encompassed diverse topics addressed in the STEADI educational program, including awareness of safe footwear selection and appropriate shoe characteristics. This is notable given the high prevalence of unsuitable footwear use among older adults, particularly women, with studies demonstrating that three-quarters of older adults wear inappropriate footwear and that older women have distinctly broader and flatter feet than their younger counterparts [21,22]. Moreover, the intervention included educational information on foot pain and the importance of routine foot examinations. There is a strong association between foot pain and recurrent falls, particularly in severe pain [23]. Geriatric practice and the STEADI framework recommend routine foot assessments to enable early detection of sensory deficits, structural abnormalities, and footwear-related problems [24].

The findings also highlighted the importance of awareness regarding postural hypotension. Previous research has identified this as an independent fall risk factor, with evidence showing an increased risk even after adjusting for confounding variables [25]. In this study, knowledge of postural hypotension improved among participants in the intervention group, suggesting that the educational program may have contributed to increased awareness of this fall-related risk factor. Additionally, the participants demonstrated improved understanding of prevention strategies, such as chair-based exercises to strengthen the muscles. This increase in knowledge is consistent with evidence from a systematic review reporting that 65% of older adults recognize the role of exercise in maintaining muscle function [26]. Knowledge regarding accurate performance and the fact that no special equipment is required to perform the chair-rise exercise also increased, although baseline knowledge was lower than that in other studies [27].

Following the intervention, knowledge of medication management improved. With this enhanced comprehension, older adults can understand how to use medications properly, identify potential side effects, and discuss with healthcare providers if they feel that the medication is disrupting their balance. It is vital to educate older adults and their caregivers on the effects of medication and the possibility of falls [28]. Consequently, educational interventions focused on medication management are vital to comprehensive fall prevention strategies.

Furthermore, the intervention group demonstrated enhanced awareness of environmental risk factors, including inadequate lighting and suboptimal flooring conditions. It is essential to educate individuals about potential fall risk factors in their home environment [29].

The findings of the present study are consistent with previous research showing that educational interventions may improve fall prevention knowledge among older adults. A study conducted in Korea reported significant improvements in fall prevention knowledge, preventive behaviors, and self-efficacy among older adults who participated in an educational program compared with a control group. The authors attributed these outcomes to the use of active and engaging educational strategies, including real-life case illustrations and videos, rather than passive instructional methods [30]. Similarly, the improvement observed in the current study may be related to the structured and interactive nature of the intervention, particularly the use of the Ask–Tell–Ask technique, which encouraged participants engagement, individualized education, and confirmation of understanding.

A significant improvement in knowledge scores was observed among participants who relied on family members for support in daily activities compared with those who received support from nurses or domestic workers. This finding may be explained by the role of family members in daily communication regarding safety measures while providing support. Additionally, family support may increase participants’ attention to health-related information and their motivation to learn, particularly when fall prevention is perceived as directly relevant to their safety and well-being [31]. The impact of falls is not confined to older adults but also affects family members. Fall injuries also impose heavy financial burdens on older adults and their families [32]. This highlights the importance of involving family members in educational interventions targeting older adults, as they can play a supportive role in enhancing learning outcomes.

Similarly, in this study, participants who were partially or fully dependent in performing daily tasks demonstrated greater improvements in knowledge scores. This may be explained by an increased perception of vulnerability and the relevance of the intervention. Previous research has shown that older adults are more likely to engage in fall prevention strategies when they perceive the intervention as personally relevant and beneficial for maintaining independence [33].

### 4.3. Effect of the Intervention on Fall Prevention Skills

The intervention resulted in a statistically significant short-term improvement in fall prevention skills of approximately 2.2 points in the intervention group. The skills section evaluated the participants’ proficiency in selecting appropriate and safe footwear, identifying unsafe footwear, safely transitioning from a sitting to a standing position, and performing chair-based exercises. Importantly, these skills were assessed through direct observation and evaluation by the researcher rather than by relying on the participants’ self-reported perceptions. However, lower reliability coefficients were observed for the skills scale (α = 0.32); therefore, findings related to this domain should be interpreted cautiously.

Regarding the chair-rise exercise and transitioning from sitting to standing movements, previous research has demonstrated that multimodal exercise interventions, particularly those focusing on balance and functional movement, can improve physical performance and reduce the risk of falls among older adults [34].

This improvement in the skill domain may be attributed to the structured and interactive nature of the intervention for older adults, emphasizing skill acquisition and knowledge development. By incorporating demonstration-based teaching and practical chair-rise exercises, the participants were able to translate theoretical information into actionable tasks. This finding is supported by previous research suggesting that interventions that incorporate real-world tasks and engagement activities are more effective in enhancing functional outcomes [35].

Another finding of this study was that greater improvements in skills were observed among participants who reported using four or more medications. This finding is supported by a study conducted in Bahrain, which indicated that older adults with higher medication use often experience a greater medication burden and sense of vulnerability, which may increase their awareness of health risks and the need for support [36]. Such vulnerability may enhance their engagement with educational and skill-based interventions, as they perceive greater benefit from strategies aimed at improving their safety and functional ability, which can relieve the feeling of burden.

### 4.4. Effect of the Intervention on Behavior and Behavioral Intentions

Importantly, our assessment of behavior and behavioral intention varied across the study groups because of the nature of the intervention. In the control group, actual behavior was measured at both the pre- and posttest stages. However, in the intervention group, the focus was on assessing behavioral intentions rather than observed behavior during these stages. This approach was chosen because immediate changes in actual behavior are unlikely to be detected after a short-term educational intervention. Instead, behavioral intention serves as a suitable proximal outcome, indicating participants’ readiness and motivation to adopt preventive practices [37]. The use of behavioral intention as an outcome measure is supported by the theory of planned behavior, which proposes that intention represents a key determinant of future behavioral adoption. Therefore, the present findings should be interpreted as improvements in participants’ readiness and willingness to adopt fall prevention behaviors rather than confirmation of actual behavioral change.

The current findings reveal a substantial gap in behavioral practices when it comes to communicating with physicians, as a large proportion of participants did not inform their doctors when experiencing balance problems. This pattern aligns with national surveillance data from the CDC 2023, which indicate that although older adults may perceive themselves as vulnerable to falls, fewer than half reported fall incidences to their healthcare providers, which may result in recurrent falls [38].

Notably, after receiving the intervention, the participants demonstrated a significant increase in their behavioral intentions. A greater number of participants expressed willingness to discuss fall risks, review their medication lists with their physicians during upcoming visits, and implement fall prevention measures. This shift reflects an increased awareness of health risks and is consistent with a systematic review indicating that educational interventions enhance short-term adoption of fall prevention behaviors [39].

The improvement in behavioral intention covered a range of relevant preventive actions, including the intention to inform a doctor when experiencing instability; review medications during medical visits; monitor the effects of new medications on balance; and implement environmental safety measures, such as rearranging furniture and improving home safety. In addition, the participants demonstrated an increased intention to engage in protective behaviors, such as performing muscle-strengthening exercises and moving cautiously when getting out of bed. Moreover, the participants demonstrated an increased intention to rise slowly from the bed. Previous studies have shown that dangling (sitting briefly on the edge of the bed before standing) reduces the likelihood of falls associated with orthostatic hypotension [40]. These findings suggest that the observed improvement in behavioral intention in the intervention group indicates a positive shift toward readiness to adopt fall prevention behaviors, even though actual behavioral changes and maintenance of preventive practices may require a longer follow-up assessment period to be fully adopted.

### 4.5. Implications for Practice and Fall Prevention Programs

The findings indicate that the educational materials obtained from the CDC’s STEADI website, brochures, and factsheets and used in the educational intervention were associated with significant short-term improvements in knowledge, skills, and behavioral intentions regarding fall prevention among older adults. Enhanced awareness of fall risks, safe shoe selection, better understanding of medication safety, and improved ability to perform chair-rise exercises may contribute to safer mobility practices and increased engagement in fall prevention behaviors in daily life. It also contributed to the development of easy-to-understand materials; improved practical skills such as performing the chair exercise and selecting appropriate footwear; and enhanced participants’ intentions to adopt future preventive behaviors, including engaging in muscle-strengthening exercises and reporting balance issues to healthcare providers. These findings underscore the importance of integrating preventive programs into older adults’ daily routines, particularly in community and healthcare settings. Family members may also play a supportive role in reinforcing learning outcomes and encouraging engagement in fall prevention practices. Another implication is that interventions should incorporate both cognitive and skill components, which may support short-term improvements in fall prevention among older adults.

### 4.6. Study Limitations

The reliance on convenience sampling and non-random group assignment introduces selection and self-selection biases, as participants were selected based on their willingness to join. This may have caused systematic differences between the intervention and control groups. Participants who voluntarily agreed to participate in the intervention may have been more motivated to engage in fall prevention education and behavior change strategies, which could have contributed to an overestimation of the observed intervention effects.

Furthermore, the skills subscale demonstrated low internal consistency (α = 0.32), while the behavior subscale demonstrated modest internal consistency (α = 0.50). Consequently, findings related to these outcomes should be interpreted cautiously. In addition, formal inter-rater reliability assessment was not conducted for the evaluation of observational skills, which may have introduced some variability in outcome assessment.

In addition, the study was conducted in a single community center, and the sample consisted exclusively of older women visiting the center, limiting generalizability. Therefore, the results cannot be generalized to older men or to older adults from different regions and community settings within Saudi Arabia.

Furthermore, the posttest was conducted immediately after the educational session, whereas in the control group, it was administered immediately after completion of the pretest. This immediate post-intervention assessment may primarily reflect short-term recall and immediate responses to the educational session rather than sustained knowledge retention, long-term learning, or actual behavioral change. Furthermore, the absence of a follow-up assessment constrains our ability to ascertain whether the observed improvements were retained over time or translated into actual behavioral change.

Only 43 intervention participants had complete post-intervention data available for paired analyses. Therefore, the possibility of attrition bias cannot be excluded. Subgroup analyses were exploratory in nature and may have been underpowered. Furthermore, no formal correction for multiple testing was applied; therefore, subgroup findings should be interpreted cautiously.

Behavioral intention rather than actual behavior was measured after the intervention. Although intention is a recognized predictor of future behavior, actual behavioral changes were not directly assessed.

Furthermore, the exclusion of older adults with significant cognitive impairments or severe mobility limitations may have resulted in a sample that was relatively more functionally independent. This limitation could affect the generalizability of the findings to older adults at a higher risk of falls. This consideration is particularly pertinent given that the educational intervention included chair-rise exercises, which necessitated a certain level of physical independence and mobility among participants.

## 5. Conclusions

In this study, the Arabic adaptation of the STEADI-based educational program was associated with short-term improvements in fall prevention knowledge, practical skills, and behavioral intentions among older adults. Although long-term outcomes were not assessed, the results support integrating fall prevention programs into community clubs and social services. Several recommendations are proposed for future research. First, a randomized controlled study design and objective behavioral outcome measures should be used to further evaluate the long-term impact of fall prevention educational interventions among older adults. Second, expanding the data collection period and including participants from different regions of the Kingdom, as well as larger, gender-diverse samples with additional items in the instrument, would enhance the generalizability of the findings and support the instrument’s validation.

## Figures and Tables

**Table 1:** Comparison of demographic and health characteristics between intervention and control groups.

Characteristics	Study Groups—No. (%)		*p*-Value
	Intervention	Control	
	(n = 64)	(n = 64)	
Age groups (in years)			
60–69	42 (65.6)	42 (65.6)	1
70–79	22 (34.4)	22 (34.4)	
Education level			
Illiterate	23 (35.9)	12 (18.8)	0.089
Primary	13 (20.3)	21 (32.8)	
Intermediate	11 (17.2)	8 (12.5)	
High School	12 (18.8)	12 (18.8)	
University or higher	5 (7.8)	11 (17.2)	
Health Issues (n = 58 & 55) *			
Diabetes	30 (51.7)	22 (40.0)	--
Cholesterol	7 (12.1)	11 (20.0)	
Blood pressure	32 (55.2)	23 (41.8)	
Musculoskeletal	15 (25.9)	22 (40.0)	
Cardiovascular	3 (5.2)	4 (7.3)	
Cancer	1 (1.7)	2 (3.6)	
Asthma	5 (8.6)	2 (3.6)	
Gastrointestinal	4 (6.9)	2 (3.6)	
Mental	0	2 (3.6)	
Eye conditions	2 (3.4)	1 (1.8)	
Thyroid condition	3 (5.2)	8 (14.5)	
Sleepless	1 (1.7)	1 (1.8)	
Use of Medications			
No	8 (12.5)	11 (17.2)	0.363
1–3	39 (60.9)	31 (48.4)	
4 and more	17 (26.6)	22 (34.4)	

* Multiple responses.

**Table 2:** Comparison of physical condition characteristics between intervention and control groups.

Characteristics	Study Groups		*p*-Value
	Intervention	Control	
	(n = 64)	(n = 64)	
Use of walking aids			
Yes	4 (6.3)	11 (17.2)	0.054
No	60 (93.8)	53 (82.8)	
Daily support for routine tasks			
No one	9 (14.1)	17 (26.6)	0.192
Domestic worker/Nurse	42 (65.6)	34 (53.1)	
Family member	13 (20.3)	13 (20.3)	
Level of independence			
Not independent	1 (1.6)	2 (3.1)	0.392
Partially dependent on myself for daily tasks	26 (40.6)	19 (29.7)	
Completely independent in performing daily tasks	37 (57.8)	43 (67.2)	
History of fall			
Yes	29 (45.3)	24 (37.5)	0.37
No	35 (54.7)	40 (62.5)	
Number of falls in the past year (n = 35 and 40)			
Once	15 (42.9)	16 (40.0)	0.589
Two to three times	13 (37.1)	12 (30.0)	
More than three times	7 (20.0)	12 (30.0)	
Cause of the last fall			
Slipping on water and wet surfaces	9 (25.7)	15 (37.5)	0.596
Tripping and stumbling on objects	10 (28.6)	11 (27.5)	
Stair-related falls	7 (20.0)	8 (20.0)	
Loss of balance, confusion, and dizziness	9 (25.7)	6 (15.0)	

**Table 3:** Baseline comparison of outcome variables between the intervention and control groups.

Outcome Variables (Scores)	Study Groups—Mean (SD)		t-Value	*p*-Value	95% Confidence Intervals for Difference in Mean
	Intervention	Control			
Knowledge	8.03 (3.3)	8.53 (2.7)	−0.946	0.346	(−1.54 to 0.54)
Skills	5.44 (1.4)	5.28 (1.7)	0.559	0.577	(−0.39 to 0.71)
Behavior for fall prevention	8.53 (2.5)	8.25 (2.3)	0.671	0.503	(−0.55 to 1.11)

Intervention

Control

**Table 4:** ANCOVA-adjusted comparison of knowledge and skills scores between the intervention and control groups.

Outcome	Control No.	Control Mean (SD)	Intervention No.	Intervention Mean (SD)	Mean Difference (95% CI)	Cohen’s d	Welch *p*	ANCOVA-Adjusted Effect (95% CI)	Partial Eta Squared (η^2^)
Knowledge	64	8.59 (2.66)	43	13.56 (1.03)	4.96 (4.23, 5.70)	2.3	<0.001	5.06 (4.46, 5.66); *p* < 0.001	0.73
Skills	64	5.31 (1.73)	43	7.53 (0.74)	2.22 (1.74, 2.71)	1.57	<0.001	1.87 (1.56, 2.18); *p* < 0.001	0.58

**Table 5:** Pre–post changes in outcome scores across participant characteristics in the intervention group.

Characteristics	Knowledge Scores		Skills Scores	
	Mean (SD)	*p*-Value	Mean (SD)	*p*-Value
Age groups (in years)				
60–69	−5.14 (3.4)	0.856	−1.90 (1.4)	0.5
70–79	−5.33 (3.1)		−1.60 (1.2)	
Education level				
Illiterate	−6.53 (3.6)	0.291	−1.93 (1.4)	0.696
Primary	−4.89 (2.8)		−1.89 (1.4)	
Intermediate	−5.00 (1.5)		−2.17 (1.1)	
High School	−4.44 (3.4)		−1.22 (1.3)	
University or higher	−3.00 (2.7)		−1.75 (1.7)	
Use of medications				
No	−4.12 (2.9)	0.585	−1.87 (1.4)	0.028
1–3	−5.46 (3.6)		−1.42 (1.1)	
4 and more	−5.44 (2.2)		−2.78 (1.5)	
Daily support for routine tasks				
No one	−4.43 (2.6)	<0.0001	−1.86 (1.3)	0.943
Domestic worker/Nurse	−4.52 (2.5)		−1.80 (1.2)	
A family member	−10.60 (3.3)		−1.60 (2.4)	
Independent level				
Not independent and partially dependent on myself for daily tasks	−6.37 (3.8)	0.034	−1.37 (1.3)	0.065
Completely independent in performing daily tasks	−4.29 (2.3)		−2.12 (1.2)	
History of fall				
Yes	−4.70 (1.9)	0.34	−2.10 (1.4)	0.16
No	−5.65 (4.0)		−1.52 (1.3)	
Number of falls in the past year				
Once	−4.72 (1.6)	0.572	−2.10 (1.2)	0.395
Two to three and more than three times	−5.37 (3.6)		−1.68 (1.4)	

**Table 6:** Post-intervention behavioral intention scores across participant characteristics in the intervention group.

Characteristics	Behavior Intention Scores	
	Mean (SD)	*p*-Value
Age groups (in years)		
60–69	13.17 (1.9)	0.231
70–79	13.8 (0.41)	
Education level		
Illiterate	13.67 (0.6)	0.159
Primary	12.22 (3.3)	
Intermediate	13.67 (0.8)	
High school	14.00 (0.0)	
University or higher	13.20 (0.8)	
Use of medications		
No	12.62 (2.8)	0.343
1–3	13.52 (1.4)	
4 and more	13.67 (0.7)	
Daily support for routine tasks		
No one	12.86 (3.0)	0.628
Domestic worker/nurse	13.45 (1.4)	
A family member	13.67 (0.5)	
Level of independence		
Not independent and partially dependent on myself for daily tasks	13.80 (0.4)	0.155
Completely independent in performing daily tasks	13.10 (2.1)	
History of fall		
Yes	13.71 (0.6)	0.206
No	13.10 (2.1)	
Number of falls in the past year		
Once	13.75 (0.6)	0.371
Two to three and more than three times	13.25 (1.9)	

**Table 7:** Pre–post comparison of knowledge and skills mean scores in the intervention group.

Outcome	n	Pre Mean (SD)	Post Mean (SD)	Mean Paired Change (95% CI)	Paired t	*p* Value	Cohen’s d
Knowledge	43	8.35 (3.33)	13.56 (1.03)	5.21 (4.22, 6.20)	10.59	<0.001	1.61
Skills	43	5.74 (1.22)	7.53 (0.74)	1.79 (1.38, 2.20)	8.78	<0.001	1.34

## Data Availability

The data presented in this study are available in Figshare at https://doi.org/10.6084/m9.figshare.32134930. The data is fully anonymized, and no identifiable participant information is included, in accordance with IRB requirements.
